# Seminal plasma hexosaminidase in patients with normal and abnormal spermograms

**Published:** 2015-09

**Authors:** Renata Julia Menendez-Helman, Claudia Sanjurjo, Patricia Vivian Miranda

**Affiliations:** 1 *Instituto de Biología y Medicina Experimental – CONICET. Buenos Aires, Argentina.*; 2 *IQUIBICEN (FCEyN, UBA – CONICET). Buenos Aires, Argentina.*; 3 *Fertilab Buenos Aires, Argentina.*; 4 *Instituto de Agrobiotecnología Rosario (INDEAR), Santa Fe, Argentina.*

**Keywords:** *Glycosidases*, *Seminal plasma*, *Hexosaminidase*, *N-acetylglucosaminidase*, *Semen Analysis*

## Abstract

**Background:**

Glycosidases profusion in male reproductive fluids suggests a possible relationship with sperm function. Although Hexosaminidase (Hex) is the most active glycosidase in epididymal fluid and seminal plasma, as well as in spermatozoa, Glucosidase is considered a marker for epididymal function and azoospermia.

**Objective:**

The aim of this study was to determine Hex activity in seminal plasma from patients with normal and abnormal spermograms and analyze its correlation with seminal parameters.

**Materials and Methods:**

In this cross sectional study, seminal plasma from azoospermic, asthenozoospermic, teratozoospermic, and normozoospermic patients was analyzed for the activity of: total Hex, HexA isoform, and glucosidase. Besides, hexosamine levels were determined, and the amount of Hex was quantified by immunoblot with a specific antibody. Correlation of Hex activity with seminal parameters was also analyzed.

**Results:**

Hex activity, like glucosidase, was significantly reduced in azoospermic samples (44, 49, and 60% reduction for total Hex, HexA and glucosidase, respectively). A reduced amount of Hex in azoospermic samples was confirmed by western immunoblot. Hex activity was negatively correlated with round cells in azoospermic samples and positively correlated with motility in asthenozoospermic ones.

**Conclusion:**

The results suggested that Hex activity was reduced in azoospermic samples and this was due to a lower amount of enzyme. The correlation to seminal parameters related to particular pathologies suggests a possible relationship of Hex with fertilizing capacity.

## Introduction

Mammalian spermatozoa acquire motility and fertilizing capacity through a complex sequence of post-testicular modification processes that start with epididymal maturation. During this phase, spermatozoa undergo biochemical and structural changes involving epididymal functions. After maturation, sperm are stored in the distal portion of the epididymis until ejaculation, at which time both cells and epididymal fluid are released and combined with secretions from accessory glands.

Some seminal plasma components are specifically produced by one organ or gland and can then be used to monitor its function. Accordingly, citric acid, fructose and glucosidase are regularly used as specific markers of prostate, seminal vesicle and epididymal functions, respectively.

Glucosidase (Glu) is one of several glycosidases, which are particularly abundant in the mammalian reproductive tract. Moreover, the epididymis seems to be the richest source of these enzymes (1). Hexosaminidase (Hex) cleaves terminal N-acetylglucosamine (GlcNAc) and N-acetylgalactosamine (GalNAc) residues. Consequently, this enzyme is often referred to as N-acetylglucosaminidase, since its activity against GlcNAc is the one regularly measured. Hex exists in two main isoforms, A and B, which share many similarities but there is difference in their specificity against a sulfated substrate that can only be hydrolysed by HexA (2).

Glu was initially reported to be the only glycosidase showing a reduced activity in seminal plasma from azoospermic men (3). However, Hex was later found to show the same pattern of activity such as Glu in relation to azoospermia and its origin (4). In addition, Hex is the most active enzyme in epididymal fluid, seminal plasma and spermatozoa (5, 6) The previous studies from our laboratory suggested its participation in epididymal maturation and sperm-zona pellucida interaction (7-10). The leading cause of infertility and sub-fertility in men is still poorly understood. A number of different studies have attempted to shed more light on the issues and defects that underlie this problem identifying of proteins associated with male fertility (11, 12). Taking into account these results, the present study aimed to determine whether the Hex found in seminal plasma was related to seminal parameters in normal or abnormal spermograms.

## Materials and methods

This cross sectional study was carried out at the Instituto de Biologiay Medicina Experimental, Buenos Aires, Argentina from March 2007 to October 2008. Remains of semen samples after spermogram analysis were provided by Fertilab after having informed consent from the patients for their exclusive use of analytical determinations.

**Materials**

All reagents used were of the highest purity or analytical grade, and were purchased from Sigma (St Louis, MO, USA), Fisher (Fairlawn, NJ, USA), Merck (Darmstadt, Germany), or J.T. Baker (Phillipsburg, NJ, USA). Polyclonal antibody against N-acetylglucosaminidase (anti-Hex) was from Nordic Immunological Laboratories (Tilburg, The Netherlands).

**Samples**

Semen was obtained by masturbation after two to seven days of sexual abstinence. An aliquot of seminal plasma was freed of sperm by centrifugation for 10 min at 3000xg, and kept frozen until use. Seminal parameters were determined following the WHO guidelines (13). Samples were classified according to the parameters listed in Table I and only those presenting single pathologies were included in this study (9 azoospermic, 8 asthenozoospermic and 7 teratozoospermic) and 9 normozoospermic samples. No information about the nature (obstructive or non-obstructive) of azoospermia was available at the time of the study. However, samples included in this work did not show some features usually associated with obstructive azoospermia, e.g. low semen volume (14), decreased or absent seminal fructose (14), absence of immature germ cells (peroxidase negative) (15). Samples having more than one seminal parameter outside the normal range were excluded from the study. 

**Enzyme activity**

Specific fluorometric substrates were used to measure the catalytic activity of total Hex (4- methylumbelliferyl- N- acetyl- β- D glucosaminide), HexA isoform (4-methylumbelliferyl-7-(sulfo- 2- acetamido- 2-deoxy-β-D-glucopyranoside) sodium salt) and Glu (4-methylumbelliferyl--D-glucoside). The assay mixture was composed of 100 μl sodium citrate pH 4.5, 100 μl enzyme solution and 100 μl substrates (1 and 2 mM for Hex and Glu, respectively). The reaction mixture was incubated 30 min at 37^°^C and stopped by the addition of 1 ml 0.5 M sodium carbonate, pH 11.3. The quantity of the fluorescent product, methylumbelliferone (MU), was measured in a Hoeffer TKO 100 fluorimeter (Hoeffer Scientific Instruments, CA, USA; emission at 380 nm and detection at 460 nm). A calibration curve was plotted using different concentrations of MU, and the enzyme activity was determined by interpolation. Enzyme activity was expressed as described below. Hex inhibition by hexosamines was determined by adding different concentrations (0, 0.03, 0.1, 0.3, 1, 3, 10, 30 and 100 mM) of GlcNAc or GalNAc to the assay. This analysis was carried out using dialyzed seminal plasma from fertile donors in triplicate. Samples were dialyzed overnight against 10 mM Tris, pH 6.8, containing 1 mM PMSF.

**Quantification of hexosamines in seminal plasma**

The concentration of hexosamines in seminal plasma was determined using the Morgan-Elson method (16). Briefly, 250 μl of seminal plasma previously diluted 1/8 were supplemented with 50 μl of 0.2 M K_2_B_4_O_7_.4H_2_O and incubated 3 min at 100^º^C. After cooling rapidly to room temperature, 1.5 ml of 6.7 mM 4-(N,N-dimethylamino)-benzaldehyde in acetic acid were added and samples were incubated 20 min at 37^º^C. Calibration curves were plotted using GlcNAc or GalNAc (0 to 3 mM) and absorbance read at 585 nm.

**Electrophoresis and Western blotting**

Following quantification by Bradford, equal amounts of total protein (15 μg) from the different seminal plasma samples were diluted in lysis buffer (50 mM Tris, 1% SDS, 5% -mercaptoethanol) and boiled for 5 min. Samples were then subjected to denaturing electrophoresis on a 10% polyacrylamide gel and transferred to PVDF membranes. Equivalent loading in each lane was verified by protein staining with 0.1% Ponceau in 5% acetic acid. Following blocking with 10% gelatin in PBS containing 0.1% Tween 20 (PBS-T), membranes were incubated overnight with anti-Hex (1/1000 in PBS-T supplemented with 1% gelatin) at 4^º^C. Membranes were then washed with PBS-T, and incubated 1 hour with peroxidase-conjugated anti-rabbit-IgG (1/10000 in PBS-T containing 1 mg/ml BSA) at room temperature. Immune complexes were located using the ECL detection system (Amersham Pharmacia Biotech Inc., NJ, USA). Quantification was done by densitometry using the ImageQuant software (Amersham Biosciences, CA, USA).

**Expression of results**

Enzyme activity was expressed in three different ways: as the total activity in the ejaculate (At=μM MU/ejaculate/min), as specific activity (Ae=nM MU/μg protein/min) and per unit of volume (Al=nM MU/μl seminal plasma/min). 

**Ethical considerations**

The present work was carried out in accordance with the code of Ethics of the World Medical Association (Declaration of Helsinki) for experiments involving human. Surplus semen samples subjected to spermogram analysis were used with the informed consent of the patients.

**Statistical analysis**

Statistical analysis was performed using the GraphPad Prism 4.0 (GraphPad Software, San Diego, CA, USA), using the Kruskal-Wallis non-parametric test followed by the Dunn’s tests for multiple comparisons or Mann Whitney non-parametric t test. Correlation analyses were performed using the Spearman test. A p-value <0.05 was considered statistically significant.

## Results

The characteristics of the samples included in this study are detailed in Table I. Only those parameters related to the pathology defining each group showed significant differences with the normozoospermic samples: sperm concentration in azoospermic, motility in asthenozoospermic and normal forms in teratozoospermic samples (Table I).

**Table I:** Criteria used for sample classification and average seminal parameters in the different groups of samples

		**Concentration (10** ^6 ^ **sperm/ml)**	**Motility Grades a+b/Grade a**		**Morphology (% normal forms)**
**Average seminal parameters in different groups**	NOR(1)	71 9	55 2	35 3	19 2
	AZO(2)	0 ***	-----	-----	-----
	AST(3)	62 10	30 5 **	7 3 ***	16.0 0.5
	TER(4)	72 7	59 2	36 2	8 1 ***

(1) Normozoospermic samples (n=9) are those having more than 20 x 10^6^ sperm/ml, more than 50 % of a+b or 25% of grade a motile sperm and more than 14% normal forms. (2) Azoospermic patients (n=9): no sperm cells in semen. (3) Asthenozoospermic samples (n=8): less than 50% a+b grade motile sperm or less than 25% grade “a” cells.

(4) Teratozoospermic samples (n=7): less than 14% normal sperm.

*: p< 0.05,

**: p<0.01,

***: p<0.001 vs. NOR samples. Values were expressed as mean ± SEM.

The activity from both Hex isoforms (total Hex, Hex T) and from the A isoenzyme (Hex A) exclusively, were determined using different substrates (see Materials and Methods). Additionally, given that Glu was regularly used as an epididymal marker, its activity was also measured to compare the results obtained for both Hex and Glu in the same group of samples.

Enzyme activity was expressed in different ways (per ml, per μg, and as the total activity in the ejaculate), since everyone might provide distinctive information. When catalytic activity was expressed per protein or volume units, it showed a narrow distribution. On the other hand, total activity per ejaculate displayed the greatest dispersion (data not shown), allowing any differences to be more evident. 

Clustering of the results according to sample classification, revealed differences only in the azoospermic group, where the three enzymes showed a significantly lower total activity per ejaculate (Table II). The degree of activity reduction did not seem to differ among Hex, HexA and Glu (44, 49 and 60%, respectively).

**Table II:** Average glycosidases activities in the different groups

		**NOR**	**AZO**	**AST**	**TER**
**HexT**	Aμl	99 4	83 7	95 3	102 3
	Ae	9.2 0.5	8.2 0.5	8.2 0.7	9.0 0.7
	At	413 53	233 33 *	310 55	425 84
**HexA**	Aμl	32 3	23 3	31 2	37 3
	Ae	3.0 0.3	2.2 0.2 *	2.7 0.3	3.3 0.3
	At	133 21	68 14 *	100 18	157 33
**Glu**	Aμl	11 2	6 2	10 2	9 1
	Ae	1.0 0.2	0.6 0.1	0.9 0.3	0.79 0.07
	At	43 7	17 4 *	30 8	37 7

The activity of total Hexosaminidase (Hex T), the A isoenzyme (Hex A) and glucosidase (Glu) were measured in triplicate in normozoospermic (NOR, n=9), azoospermic (AZO, n=9), asthenozoospermic (AST, n=8) and teratozoospermic (TER, n=7) samples as detailed in materials and methods. Results (mean ± SEM) were expressed as: nM MU/μl seminal plasma/min (Aμl), nM MU/μg protein/min (Ae) and μM MU/ ejaculate/min (At).

*: p<0.05 vs. NOR.

**Figure 1:**
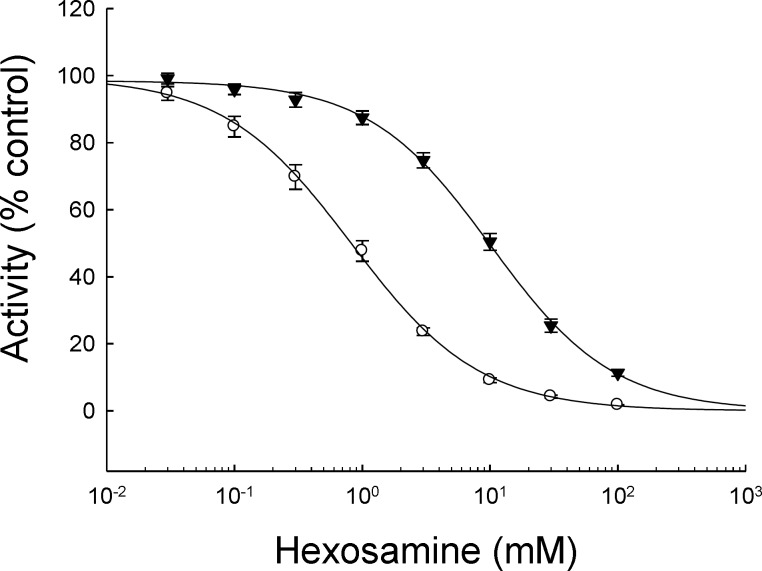
Inhibition of seminal plasma Hexosaminidase (Hex) by hexosamines. N-acetyglucosamine (GlcNAc,  ) or N-acetylgalactosamine (GalNAc,  ).

**Figure 2:**
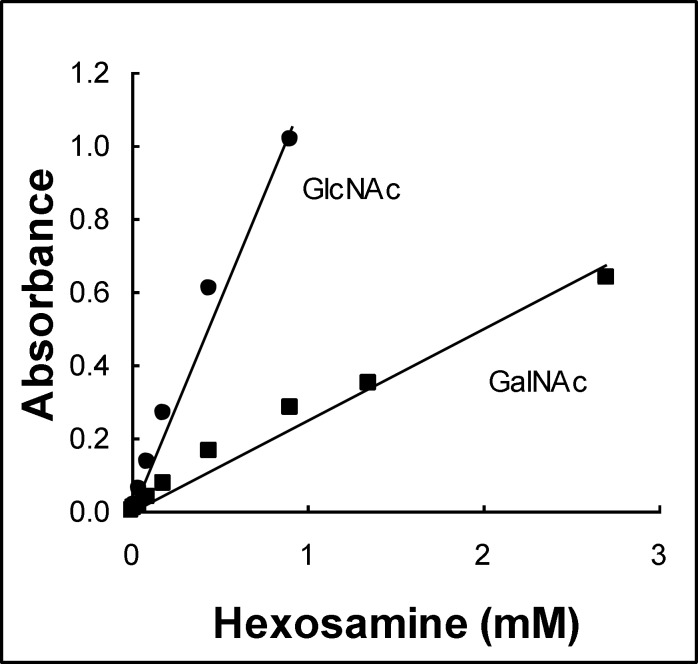
Standard curves for both hexosamines, N-acetyglucosamine (GlcNAc) and N-acetylgalactosamine (GalNAc), in the Morgan- Elson method.

**Figure 3:**
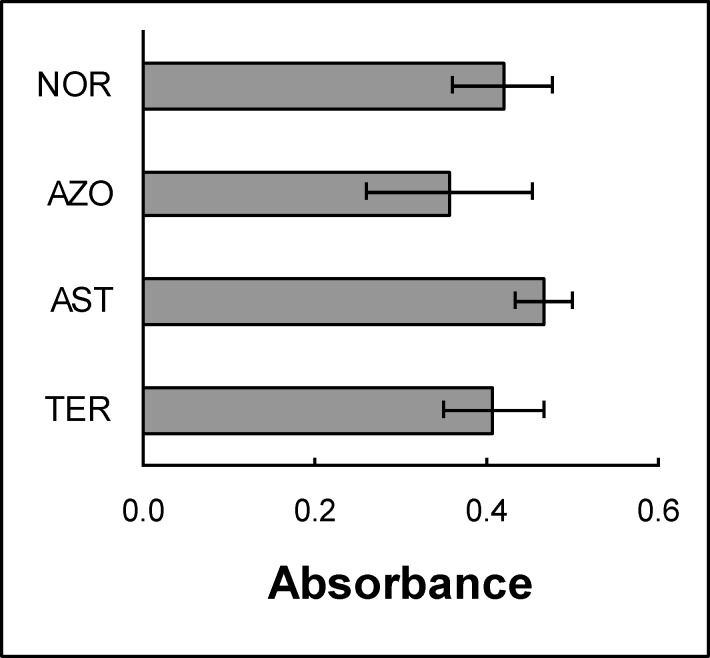
Hexosamines in seminal plasma from the different groups. Normozoospermic (NOR), azoospermic (AZO), asthenozoospermic (AST) and teratozoospermic (TER) samples.

Considering that seminal vesicles secrete hexosamines which are able to inhibit Hex catalytic capacity, we analyzed if the lower Hex activity in the azoospermic group could be due to a higher concentration of hexosamines. First, the inhibitory capacity of both hexosamines on seminal plasma Hex was analyzed using samples from fertile donors. Both monosaccharides showed typical inhibition curves with EC50 values of 10 ± 1 and 0.8 ± 0.3 mM for GlcNAc and GalNAc, respectively (Figure 1). On the other hand, the concentration of hexosamines in seminal plasma from each group was determined. This quantification was currently done using a colorimetric procedure where both monosaccharides react giving a different response (Figure 2). Although the signal could not be assigned to a particular hexosamine, the average absorbance was found to be similar for all groups (Figure 3) suggesting that different levels of these monosaccharides in seminal plasma would not be the reason for the low Hex activity in the azoospermic group. To evaluate the possibility that the reduced Hex activity was the result of a lower concentration of enzyme, the amount of enzyme in seminal plasma was determined by western immunoblot. 

**Figure 4:**
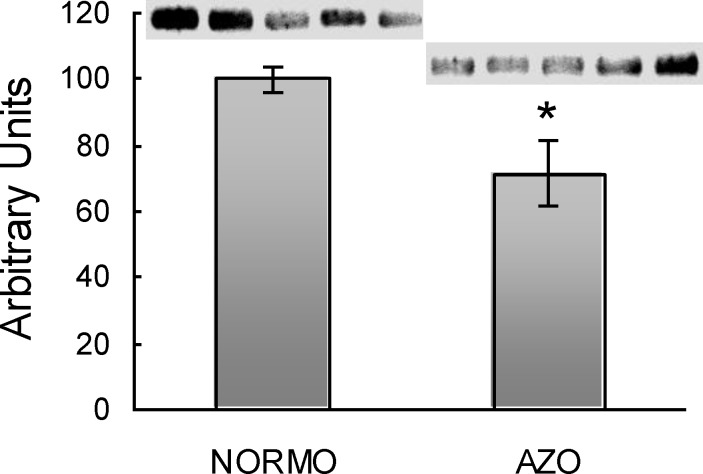
Quantification of Hex in seminal plasma from normozoospermic (NORMO) and azoospermic (AZO) samples

To normalize samples, equal amount of total protein was loaded in each lane and corroborated after transfer by total protein staining. The signal was significantly reduced in the azoospermic group (p=0.02) when compared to normozoospermic samples (Figure 4).

In order to evaluate whether Hex activity in seminal plasma was related to fertility capacity, the relationship between enzyme activity and seminal parameters was analyzed. In azoospermic samples, HexA (Aμl, At) showed a negative correlation with total round cells (r^2^=-0.81, p=0.01; r^2^=-0.69, p=0.04) and a positive correlation with acid phosphatase (Ae, r^2^=0.89, p=0.01), suggesting that the prostate would produce this Hex isoform. In the case of HexT, a strong negative correlation was found with both the concentration of total round cells (Aμl, r^2^=-0.82, p=0.01; At, r^2^=-0.74, p=0.03), as well as the peroxidase negative ones (Aμl, r^2^=-0.69, p=0.04). In the teratozoospermic group of samples, HexA (At) was correlated negatively with the concentration of peroxidase positive round cells (r^2^=-0.80, p=0.03) and Glu (Aμl) was correlated negatively with normal sperm morphology (r^2^=-0.88, p=0.01). When the asthenozoospermic group of samples was analyzed, HexT showed a strong positive correlation with motility (Aμl, r^2^=0.76, p=0.04).

## Discussion

Male fertility relies not only in the correct production of spermatozoa within the testis, but also for efficient epididymal maturation, which is necessary to provide the fertilizing ability of sperm. As a result, epidiymal function is often considered in the study of infertility, its putative treatment and the search for fertility markers.

The epididymis displays the highest glycosidase activity and, unlike other organs, these activities increase with sexual maturation, suggesting a relationship between these enzymes and sperm maturation. Although glycosidases were originally investigated to monitor epididymal function, an early report found that Glu was the only enzyme absent in cases of azoospermia (3). Although the reason for this association between reduced glycosidase activity and azoospermia has not been analyzed in detail, Glu was selected as a specific marker of azoospermia caused by duct obstruction (4). 

However, although Hex behaved like Glu in that study, it was not considered as a putative marker of epididymal function and/or azoospermia (4).

During the last years, our laboratory has been studying the possible involvement of Hex in mammalian reproduction. While analyzing Hex activity in the human epididymis, we found an increasing activity and also an interesting change in the isoform composition along the length of this organ (7). The isoenzymes ratio in the luminal fluid of the epididymal cauda was similar to the one found in sperm membrane extracts (7). Lately, it was verified the presence of Hex in the acrosomal plasma membrane, and the ability of exogenous enzyme to bind to human sperm (10). Furthermore, it was reported the involvement of Hex in sperm binding to the zona pellucida and, moreover, that sperm incubation with Hex improved its ability to bind to the ZP in cases with an originally low binding capacity (8). Additionally, Hex seems to be related to the induction of acrosome reaction by GlcNAc, which showed a positive relationship with the in vitro fertilization outcome (9, 17). Taking into account all these evidences it was hypothesized that Hex activity in seminal plasma might be related to fertility.

Hex activity was found to be similar in normo, astheno and teratozoospermic samples, but, like Glu, Hex activity was lower in cases of azoospermia. A further report on lysosomal enzymes in seminal plasma indicated reduced Hex activity in oligo and azoospermic patients (18), although the samples used in this study had altered protein and fructose concentrations, suggesting that multiple factors could be affecting enzyme activity. Later studies described a reduced Hex activity in azoospermic samples, although it was only for the A isoenzyme (19, 20). The results reported in this study agreed with these precedents since, when specific activity was analyzed, only the HexA isoform was significantly reduced. However, it was also found a positive correlation between this activity and acid phosphatase, suggesting a prostatic origin for this isoenzyme. This is equally valid at the time of finding a fertility marker in seminal plasma, and it would not be related to epididymal function. For this reason, total activity in the ejaculate (parameter used for the accepted epididymal marker Glu) was also analyzed, and it was found that total Hex showed an equivalent reduced activity in seminal plasma from azoospermic patients.

To analyze the origin of the reduced Hex activity in azoospermic samples, the present study initially focused on the fact that seminal vesicles secrete hexosamines, making seminal plasma particularly rich in sugars that cannot be metabolized by sperm, but are able to inhibit Hex (21). Then it was analyzed, if a different concentration of these monosaccharides could be the reason for the reduced Hex activity in azoospermic samples. The similar concentration of hexosamines in all the groups and the comparable effect of exogenous sugars on seminal plasma before and after dialysis (data not shown) indicated that the presence of Hex inhibitors of low molecular weight would not be the cause of the reduced Hex activity. Western blot results indicated that the drop in enzyme activity would be due to a reduced amount of Hex. This apparent contradiction between the similar Hex specific activity within all the samples and a diminished amount of enzyme in seminal plasma could be related to a Hex modulator from accessory sex glands that is currently under study in our laboratory (data not shown).

The possibility that the reduced Glu activity in cases of azoospermia was due to a similar decrease in the amount of enzyme has not been analysed yet.

Correlation analysis showed that Hex activity was related to particularly interesting seminal parameters in some of the pathological groups. In azoospermic samples, Hex was negatively correlated with total and peroxidase-negative round cells. This result indicates that Hex activity would be reduced when leukocytes and/or immature germ cells are present. The presence of these cells could be related to the azoospermia and, more importantly, it has been associated with reduced fertility (22, 23). Unexpectedly, no correlations were found for Glu in the same azoospermic group.

It should be noted that the absence of Hex of sperm origin in azoospermic samples would not be the cause for the reduced activity, since only 3% of the Hex activity in semen was associated with cells. 

In asthenozoospermic samples, Hex activity was correlated positively with the percentage of motile sperm, suggesting that the enzyme might be related to the development, maintenance or modulation of motility. These possibilities are currently under study in our laboratory.

It is interesting to note that, although the Hex activity is particularly high in epididymal fluid and semen a catalytic task for this enzyme seems unlikely. Hex is an acidic glycosidase, which is almost inactive at physiological pH. In fact, N-acetylglucosamine residues on sperm were highest in the distal epididymis, where Hex activity reached its maximum (7, 24). Moreover, the presence of N-acetylgucosamine residues in the human sperm surface has been positively correlated with fertilizing ability (25).

Although some interesting features were found when analyzing Hex activity in seminal plasma, its relationship with fertility capacity would require the study of the sperm-associated enzyme. Irregularities in sperm Hex activity were reported for oligoasthenoteratozoospermic patients (26). However, the origin, subcellular location and significance of these abnomalities remained to be established.

Recent evidence from our laboratory supported the presence of Hex at the human sperm plasma membrane and the ability of exogenous human recombinant enzyme to bind to sperm (10). Given that this treatment could improve binding to the zona pellucida (8), future efforts should be focused on the relationship between sperm plasma membrane Hex and fertility capacity.

## Conclusion

In conclusion, the findings showed a significantly lower Hex activity in seminal plasma obtained from azoospermic patients. Further analysis confirmed that different levels of hexosamines in seminal plasma would not be the reason for the low Hex activity in the azoospermic group, but instead to a reduced amount of enzyme. Finally, the correlation of Hex activity with parameters related to particular pathologies suggests a possible relationship with fertilizing capacity.

## Note

***This article was extracted from student’s thesis. (Renata J. Menendez-Helman)***
